# Tetratricopeptide repeat domain 9A modulates anxiety-like behavior in female mice

**DOI:** 10.1038/srep37568

**Published:** 2016-11-21

**Authors:** Lee Wei Lim, Smeeta Shrestha, Yu Zuan Or, Shawn Zheng Kai Tan, Hwa Hwa Chung, Yang Sun, Chew Leng Lim, Sharafuddin Khairuddin, Thomas Lufkin, Valerie Chun Ling Lin

**Affiliations:** 1 School of Biomedical Sciences, Li Ka Shing Faculty of Medicine, the University of Hong Kong, Hong Kong, China; 2 School of Biological Sciences, Nanyang Technological University, Singapore; 3 Department of Biology, Clarkson University, Potsdam, New York, USA

## Abstract

Tetratricopeptide repeat domain 9A (TTC9A) expression is abundantly expressed in the brain. Previous studies in TTC9A knockout (TTC9A^−/−^) mice have indicated that TTC9A negatively regulates the action of estrogen. In this study we investigated the role of TTC9A on anxiety-like behavior through its functional interaction with estrogen using the TTC9A^−/−^ mice model. A battery of tests on anxiety-related behaviors was conducted. Our results demonstrated that TTC9A^−/−^ mice exhibited an increase in anxiety-like behaviors compared to the wild type TTC9A^+/+^ mice. This difference was abolished after ovariectomy, and administration of 17-β-estradiol benzoate (EB) restored this escalated anxiety-like behavior in TTC9A^−/−^ mice. Since serotonin is well-known to be the key neuromodulator involved in anxiety behaviors, the mRNA levels of tryptophan hydroxylase (TPH) 1, TPH2 (both are involved in serotonin synthesis), and serotonin transporter (5-HTT) were measured in the ventromedial prefrontal cortex (vmPFC) and dorsal raphe nucleus (DRN). Interestingly, the heightened anxiety in TTC9A^−/−^ mice under EB influence is consistent with a greater induction of TPH 2, and 5-HTT by EB in DRN that play key roles in emotion regulation. In conclusion, our data indicate that TTC9A modulates the anxiety-related behaviors through modulation of estrogen action on the serotonergic system in the DRN.

Mood and anxiety disorders are the most common mental illnesses with a lifetime prevalence as high as 20%^1^,^2^. It has been reported that women have a higher rate to develop this disorder with twice the risk than men^3^. In particular, the higher incidence of female suffering from mood and anxiety disorder is mainly related to hormonal cycle changes, family responsibility, work pressure, and comorbidity from somatic illnesses^4^,^5^. Although no specific etiology that causes mood and anxiety has been identified, it is likely that genetic, biological, environmental, and lifestyle factors may play a major role in its pathophysiology.

Mood and anxiety disorders can be treated effectively by either medication, usually targeting the serotoninergic (5-hydroxytryptamine, 5-HT) system, and/or psychotherapy. The dysfunction of 5-HT neurotransmission has long been associated with major depressive disorder, anxiety, and suicidal behavior^6^,^7^. In clinical and experimental studies, pathological alterations of the amino acid tryptophan hydroxylase (TPH, a precursor for the biosynthesis of serotonin) and the 5-HT transporters (5-HTT) have been reported to either functionally increase or reduce 5-HT neurotransmission in specific area of the limbic and frontal regions^7^,^8^,^9^. Of particular interest, reduction of TPH and 5HTT in ventromedial prefrontal cortex (vmPFC) and increased 5-HTT in dorsal raphe nucleus (DRN) is associated with mood- and anxiety-like behavior in animal models^8^,^9^.

Tetratricopeptide repeat domain 9A (TTC9A) was first isolated from a brain cDNA library^10^, and it shares significant homology with FK506 Binding Proteins (FKBP) cyclophilin 40, FKBP51, FKBP52 and FKBP38 that are steroid receptor co-chaperones involved in modulating steroid receptor assembly and maturation^11^,^12^. TTC9A protein contains 3 tetratricopeptide repeat (TPR) domains that are 34 amino acid consensus motif present in varying numbers in a large family of TPR-containing proteins^11^,^13^. These TPR motifs in tandem arrays form antiparallel α-helical hairpins that serve as an important protein interaction interface and are involved in regulating diverse biological processes including steroid receptor signaling^11^,^14^.

TTC9A is expressed in the mouse embryonic stem cells^15^. *In situ* hybridization study of mouse embryos at day 13.5 showed abundant TTC9A expression only in the neural tissues (Fig. 1). TTC9A protein is ubiquitously expressed in all tissues after birth, with the highest expression in the brain and the lowest expression in the liver^15^,^16^. TTC9A expression is regulated by both estrogen and progesterone in breast cancer cells. It is drastically induced by progesterone in progesterone receptor-transfected breast cancer cells MDA-MB-231 which also exhibited^16^, growth inhibition and focal adhesion in these cells^17^,^18^; its expression is down-regulated by the estrogen in MCF7 breast cancer cells^16^. These data seem to suggest a negative association between TTC9A expression, cell growth and the involvement of TTC9A in ovarian steroid hormone signaling in human breast cancer cells. However, studies in mice revealed that estrogen significantly up-regulated the TTC9A expression in estrogen target tissues such as the uterus and mammary gland^15^.

Estrogen has been implicated in the regulation of emotion within the limbic system^19^. Earlier studies have consistently shown that the fluctuation of estrogen during puberty, pregnancy, and pre-or post-menopause periods increased the risk of developing mood and anxiety disorders, and cognitive dysfunction^20^. TTC9A has been shown to negatively regulate estrogen activity in TTC9A knockout (TTC9A^−/−^) mice^15^,^21^. In this study, we investigated the role of TTC9A on mood- and anxiety-related behaviors using the TTC9A^−/−^ mice model. A battery of behavior tests on anxiety, memory function, social behavior, hedonia, and behavioral despair were conducted. The study found that TTC9A^−/−^ female mice were more anxious as compared to wildtype (TTC9A^+/+^) mice and the phenotype appears to be due to the difference in response to 17-β-estradiol benzoate (EB) treatment. The anxious phenotype in TTC9A^−/−^ mice is associated with greater induction of TPH2 and 5-HTT by EB administration that positively influences the serotonergic system. These findings indicate for the first time that TTC9A modulates anxiety-related behaviors through negative regulation of estrogen action on serotonergic system in the DRN.

## Results

### TTC9A^−/−^ mice exhibit increased anxiety-like behaviors

**Figure 2:**
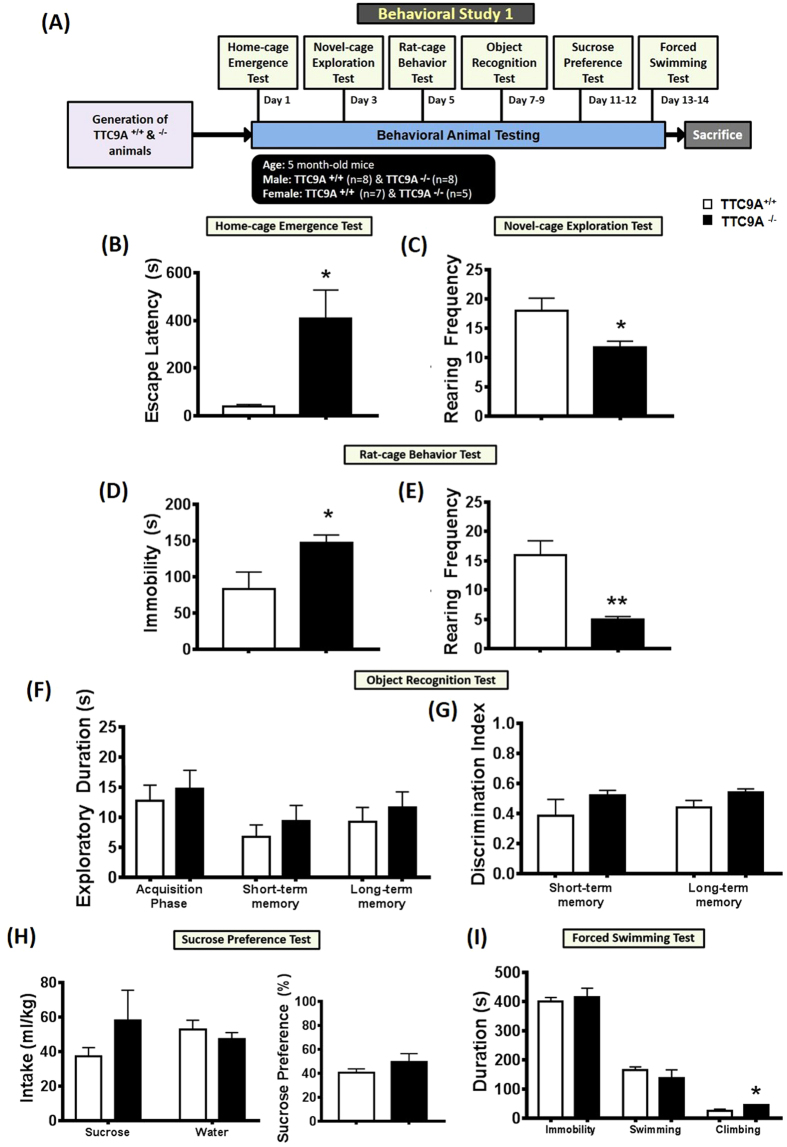
Behavioral phenotypes characterization in TTC9A^−/−^ female mice. A schematic diagram represents the time-line of the experimental design for behavioral study 1 (**A**). Effects of TTC9A^−/−^ on a set of behavioral battery testing for anxiety-related and exploratory behaviors ((**B**) home-cage emergence test; (**C**) novel-cage exploration test; (**D**,**E**) rat-cage exploration test); memory function ((**F**,**G**) object recognition test); hedonia ((**H**) sucrose preference test); and behavioral despair (**I**, forced swimming test) in female mice. Note. The deletion of TTC9A gene induced anxiety-like behavior; and no significant difference were found in the memory function, hedonia, and behavioral despair measures. The data were presented as mean ± S.E.M. Indication: *Represents *p* < 0.05, **Represents *p* < 0.01.

**Figure 3:**
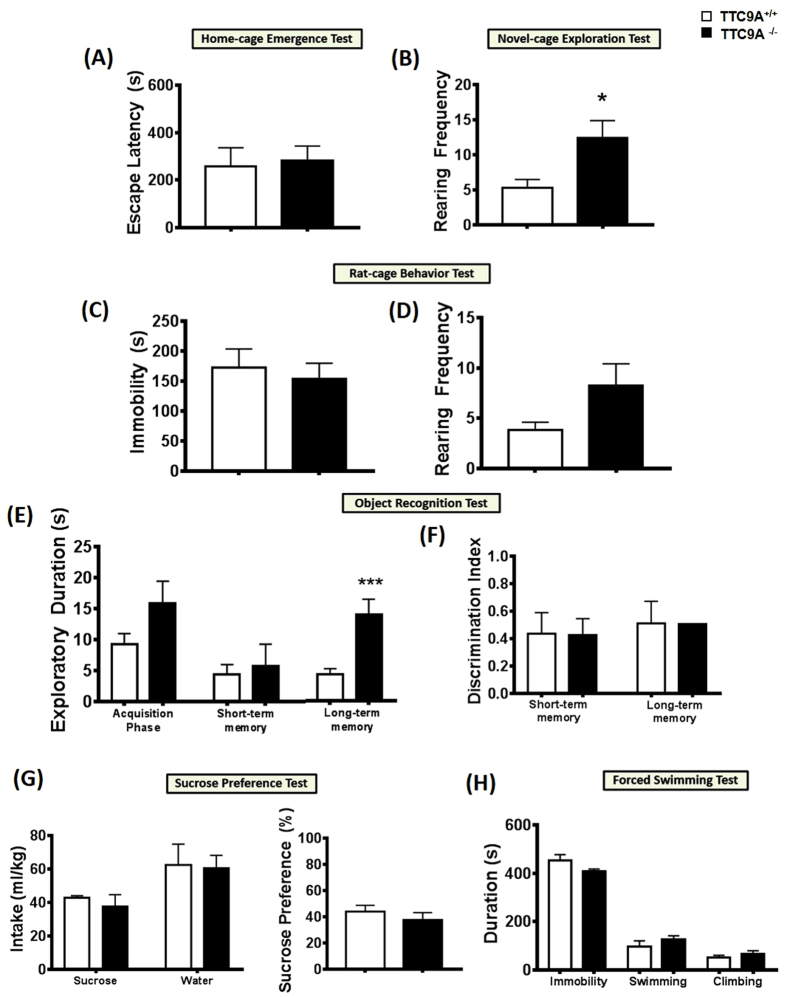
Behavioral phenotypes characterization in TTC9A^−/−^ male mice. Effects of TTC9A^−/−^ on a set of behavioral battery testing for anxiety-related and exploratory behaviors ((**A**) home-cage emergence test; (**B**) novel-cage exploration test; (**C**,**D**) rat-cage exploration test); memory function ((**E**,**F**) object recognition test); hedonia ((**G**) sucrose preference test); and behavioral despair ((**H**) forced swimming test) in male mice. Note, despite TTC9A^−/−^ increased rearing behavior (marginal effect; *p* = 0.041) in the novel-cage exploration test, no significant differences were found in the overall anxiety, memory function, hedonia, and behavioral despair measures. The data were presented as mean ± S.E.M. Indication: *Represents *p* < 0.05, ***Represents *p* < 0.005.

In the anxiety tests, there were significant effects of increased latency of TTC9A^−/−^ female mice to escape from their home-cage (t_(8)_ = 3.113, *p* = 0.014; Fig. 2B) in the home-cage emergence test, and increased immobility (t_(7)_ = 4.942, *p* = 0.002; Fig. 2D) in the rat-cage behavior test as compared to the TTC9A^+/+^ female mice. In addition, a reduction of exploratory rearing was consistently found in both the novel-cage exploratory test (t_(7)_ = −2.408, *p* = 0.047; Fig. 2C) and rat-cage behavior test (t_(7)_ = −5.043, *p* = 0.001; Fig. 2E) of TTC9A^−/−^ female mice as compared to the TTC9A^+/+^ animals. On the other hand, analysis of the male mice showed no significant difference in the escape latency of home-cage emergence test (t_(13)_ = 0.245, *p* = n.s.; Fig. 3A), immobility (t_(13)_ = −0.492, *p* = n.s.) and rearing (t_(13)_ = 1.815, *p* = n.s.) behaviors in the rat-cage behavior test (Fig. 3C,D) between the TTC9A^−/−^ and TTC9A^+/+^ animals. However, a slight increase of rearing behavior (t_(12)_ = 2.292, *p* = 0.041) was found in the TTC9A^−/−^ male mice in the novel-cage exploration test (Fig. 3B).

### TTC9A knockout did not affect memory function

In the object-recognition test, a repeated-measures analysis showed no significant difference between TTC9A^+/+^ and TTC9A^−/−^ mice for group (F_(1,8–10)_ < 3.371, *p* = n.s.) and their interaction group × exploration level (F_(1,8–10)_ < 1.158, *p* = n.s.) in both the female and male mice (Figs 2F and 3E). In the acquisition phase, there were no differences of exploration levels between the TTC9A^−/−^ and TTC9A^+/+^ of both the female and male mice (t_(10–12)_ < 1.673, *p* = n.s.). For the time ratio of discrimination between the novel and the familiar objects, no significant effects were found in both the TTC9A^−/−^ female and male (short-term memory phase: t_(10–12)_ < 1.016, *p* = n.s.; long-term memory phase: t_(10–12)_ < 1.606, *p* = n.s.) mice as compared to the TTC9A^+/+^ animals (Figs 2G and 3F).

### TTC9A^−/−^ mice did not exhibit hedonism

In the sucrose preference test, TTC9A gene deletion had no significant effect on the amount of sucrose intake (t_(10)_ < 1.297, *p* = n.s.), level of water consumption (t_(10–12)_ < −0.149, *p* = n.s.), and percentage of sucrose preference (t_(10–12)_ < 1.322, *p* = n.s.) in both the female and male mice of TTC9A^−/−^ and TTC9A^+/+^ animals (Figs 2H and 3G). In addition, there was no difference in the average body weight (t_(10–12)_ < 0.852, *p* = n.s.) between the TTC9A^−/−^ and TTC9A^+/+^ mice of both the female (TTC9A^−/−^: 21.79 ± 0.46 g; and TTC9A^+/+^: 22.60 ± 0.40 g) and male (TTC9A^−/−^: 31.05 ± 1.03 g; and TTC9A^+/+^: 29.63 ± 1.37 g) animals.

### TTC9A^−/−^ mice did not exhibit increase in behavioral despair

In the forced swimming test, no significant changes were found in the immobility (t_(10)_ = 0.525, *p* = n.s.) and swimming in TTC9A^−/−^ female mice (t_(10)_ = −0.957, *p* = n.s.) (Fig. 2I); however, there was a marginal increase in climbing behavior (t_(7)_ = 2.389, *p* = 0.048) of the TTC9A^−/−^ female mice when compared to the TTC9A^+/+^ animals, indicating the enhancement of noradrenergic neurotransmission in mediation of anxiogenic response. In TTC9A^−/−^ male mice, no difference was found in immobility (t_(13)_ = −1.747, *p* = n.s.), swimming (t_(13)_ = 1.042, *p* = n.s.), and climbing (t_(13)_ = 0.974, *p* = n.s.) behaviors as compared to the TTC9A^+/+^ male animals (Fig. 3H).

### Effect of TTC9A^−/−^ on behavioral response to estrogen treatment

**Figure 4:**
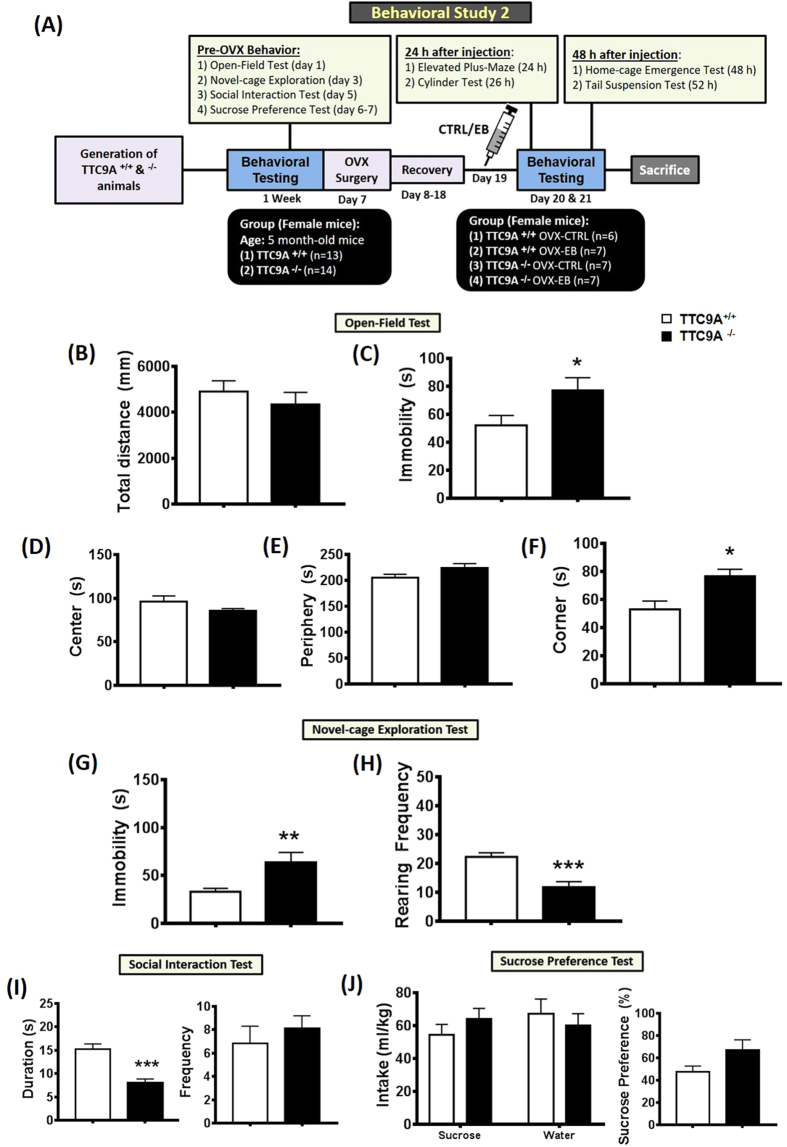
Behavioral phenotypes characterization in TTC9A^−/−^ female mice. A schematic diagram represents the time-line of the experimental design for behavioral study 2 (**A**) with manipulation of ovariectomy (OVX) procedure and 17-β-estradiol benzoate (EB) treatment. A new batch of TTC9A^−/−^ female mice were generated and tested for mood- and anxiety-related behaviors. TTC9A^−/−^ animals consistently showed anxiety in the open-field test with increased immobility duration and time-spent in the corner zone (**C**,**F**); as well as increased immobility and reduced rearing frequency in the novel-cage exploration test (**G**,**H**). Interestingly, there was an overall reduction on social behavior in the social interaction test (**I**), and no significant change was found on the sucrose preference test which measured the level of hedonia (**J**). The data were presented as mean ± S.E.M. Indication: *Represents *p* < 0.05, **Represents *p* < 0.01, ***Represents *p* < 0.005.

Before ovariectomy, the animals were tested for their anxiety-related and exploratory behaviors. In open-field test, there was no significant difference in time-spent between the TTC9A^−/−^ and TTC9A^+/+^ female mice in the center (t_(17)_ = 1.223, p = n.s.) and periphery (t_(20)_ = 1.68, *p* = n.s.) zones (Fig. 4D,E). However, TTC9A^−/−^ female mice displayed significant increase in immobility (t_(18)_ = −3.342, *p* = 0.004) and time-spent in the corner zone (t_(16)_ = −2.867, *p* = 0.011) compared to the TTC9A^+/+^ mice (Fig. 4C,F), indicating anxiogenic response. In addition, we found no significant difference in the distance moved (t_(17)_ = 0.813, *p* = n.s.) of the TTC9A^−/−^ (4334 ± 531.2 mm) and TTC9A^+/+^ (4907 ± 467.5 mm) female mice, suggesting that the immobility behavior was not due to general locomotor deficits (Fig. 4B). Furthermore, TTC9A^−/−^ mice showed a significant increase in immobility duration (t_(22)_ = −3.031, *p* = 0.006), and a decrease in rearing frequency (t_(23)_ = 4.088, *p* < 0.001) in the novel-cage exploration test (Fig. 4G,H). In the social interaction test, we found a decrease in the duration of social interaction behavior (t_(10)_ = 5.239, *p* < 0.001), but no changes were demonstrated in their interaction frequency (t_(11)_ = −0.739, *p* = n.s.) between TTC9A^−/−^ and TTC9A^+/+^ female mice (Fig. 4I). In sucrose preference test, no difference was revealed in the amount of sucrose intake (t_(24)_ = 1.037, *p* = n.s.), the level of water consumption (t_(25)_ = −0.612, *p* = n.s.), and the percentage of sucrose preference (t_(25)_ = 0.704, *p* = n.s.) in TTC9A^−/−^ mice as compared to the TTC9A^+/+^ animals (Fig. 4J). Additionally, there was also no change in the average body weight (t_(24)_ = −0.232, *p* = n.s.) of TTC9A^−/−^ (21.4 ± 0.38 g) and TTC9A^+/+^ (21.6 ± 0.46 g) female mice. Altogether, these data are in line with the behavioral study in the earlier experiment.

**Figure 5:**
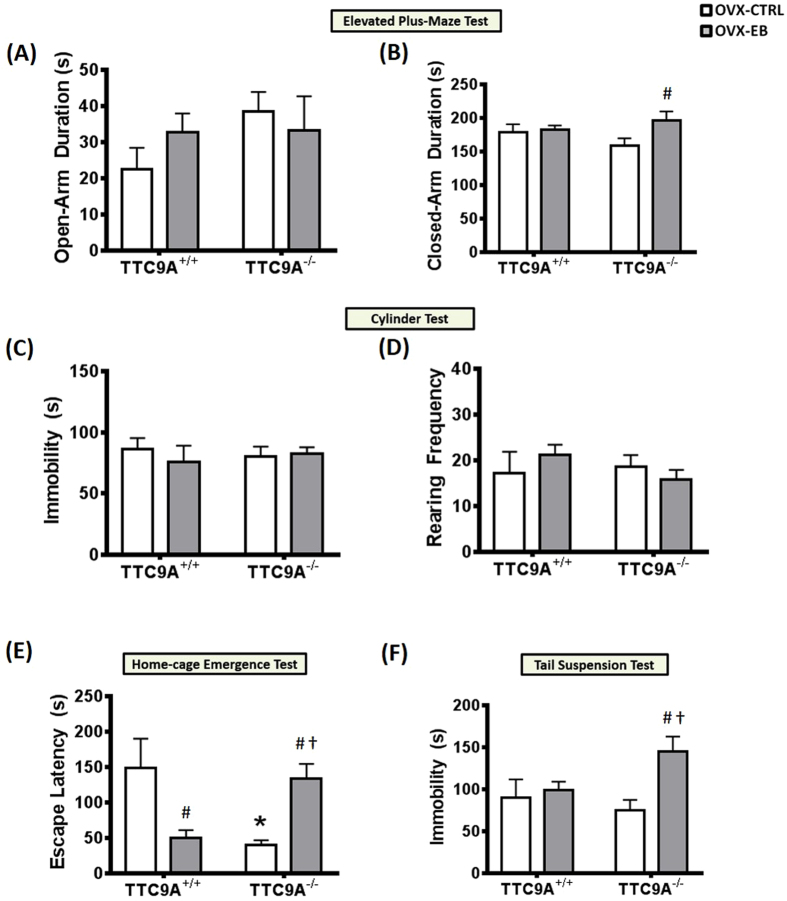
Effects of estrogen regulation on TTC9A^−/−^. A set of bar graphs showing the behavioral data for mood- and anxiety-related measures of TTC9A^−/−^ female mice after ovariectomy (OVX). Animals were injected with either 17-β-estradiol benzoate (EB) or CTRL, and subsequently tested in the elevated plus-maze (**A**,**B**), cylinder test (**C**,**D**), home-cage emergence test (**E**), and tail-suspension test (**F**) in the following time order of 24, 26, 48, and 52 h after injection. Interestingly, there were remarkable effects on anxiety (**E**) and behavioral despair (**F**) after 48 and 52 h in the TTC9A^−/−^ OVX-EB mice. In the TTC9A^+/+^ animals, EB treatment reversed the anxiety-like behavior in the home-cage emergence test, while no difference was observed in the immobility measures of tail-suspension test. The data were presented as mean ± S.E.M. Indication: ^#^Significant difference from the respective group of OVX-CTRL treated mice; *Significant difference from the TTC9A^+/+^ OVX-CTRL mice; and ^†^Significant difference from the TTC9A^−/−^ OVX-CTRL treated mice; (*p* < 0.05).

As our previous study showed the TTC9A negatively regulates estrogen action in mammary development, we tested the hypothesis whether the effect of TTC9A gene deletion on anxiety is also related to its effect on estrogen response in female mice. This would be done by examining the effect of estrogen on anxiety-like behaviors in ovariectomized (OVX) mice. After ovariectomy, mice were given a 10-day recovery period and subsequently injected with either sesame oil (CTRL) or 17-β-estradiol benzoate (EB) in sesame oil. Twenty four hours after injection, two-way ANOVA showed no significant difference in the interaction, genotype, and treatment in the duration of time-spent in the open-arm (F_(1,18)_ < 1.25, *p* = n.s.); however, TTC9A^−/−^ OVX-EB mice showed an increase in closed-arm duration (*p* = 0.02) in the elevated plus-maze (Fig. 5A,B). Analysis of the number of entries to closed or open arm showed no significant difference in interaction, genotype, and treatment (closed: F_(1,21)_ < 0.36, open: F_(1,22)_ < 2.66), all *p* = n.s.; data not shown). In cylinder test, two-way ANOVA with post-hoc analysis also revealed no significant difference in genotype (F_(1,19–20)_ < 0.412), *p* = n.s.), treatment (F_(1,19–20)_ < 0.179, *p* = n.s.) and their interactions (F_(1,19–20)_ < 1.266), *p* = n.s.) for immobility and rearing behaviors in the cylinder environment (Fig. 5C,D).

Forty eight hours after injection, in the home-cage emergence test, two-way ANOVA revealed a significant difference in interaction (F_(1,21)_ = 17.05, *p* = 0.0005), but not in genotype or treatment (F_(1,21)_ = 0.29 and F_(1,21)_ = 0.01, respectively, all *p* = n.s.). Post-hoc analysis showed a significant reduction on the escape latency (*p* < 0.01) in TTC9A^+/+^ OVX-EB group as compared to the TTC9A^+/+^ OVX-CTRL animals, indicating an EB-induced anxiolytic effect in the home-cage emergence test. There was also a significant decrease of escape latency in the TTC9A^−/−^ OVX-CTRL mice compared to TTC9A^+/+^ OVX-CTRL mice (*p* < 0.01). Interestingly, we found an increase of escape latency in the home-cage emergence test in TTC9A^−/−^ OVX-EB treated mice compared to TTC9A^−/−^ OVX-CTRL and TTC9A^+/+^ OVX-EB animals (*p* < 0.05). In tail-suspension test, two-way ANOVA revealed a significant difference between treatments (F_(1,23)_ = 6.61, *p* = 0.017), but no difference between genotypes or interactions (F_(1,23)_ = 0.96 and F_(1,23)_ = 3.90, respectively, all *p* = n.s.). Post-hoc analysis showed a significant increase of tail-suspension immobility in the TTC9A^−/−^ OVX-EB treated mice as compared to the TTC9A^−/−^ OVX-CTRL (*p* < 0.01) and TTC9A^+/+^ OVX-EB (*p* < 0.05) animals. This suggests that TTC9A^−/−^ mice are susceptible to estrogen-induced anxiety-like behaviors (Fig. 5E,F).

### Effect of TTC9A gene KO on the expression of TPH and 5-HTT mRNA in TTC9A^−/−^ mice

It has been reported that EB treatment increases the expression of TPH1 and TPH2 in the dorsal raphe nucleus, which in turn regulates serotonin synthesis^22^,^23^. As serotonergic system is fundamentally important in anxiety disorders^24^,^25^, we investigated if the changes in mRNA expression levels of TPH1, TPH2, and 5-HTT in the vmPFC and the DRN of TTC9A^−/−^ mice would be the reason for increased anxiety-like behaviors.

**Figure 6:**
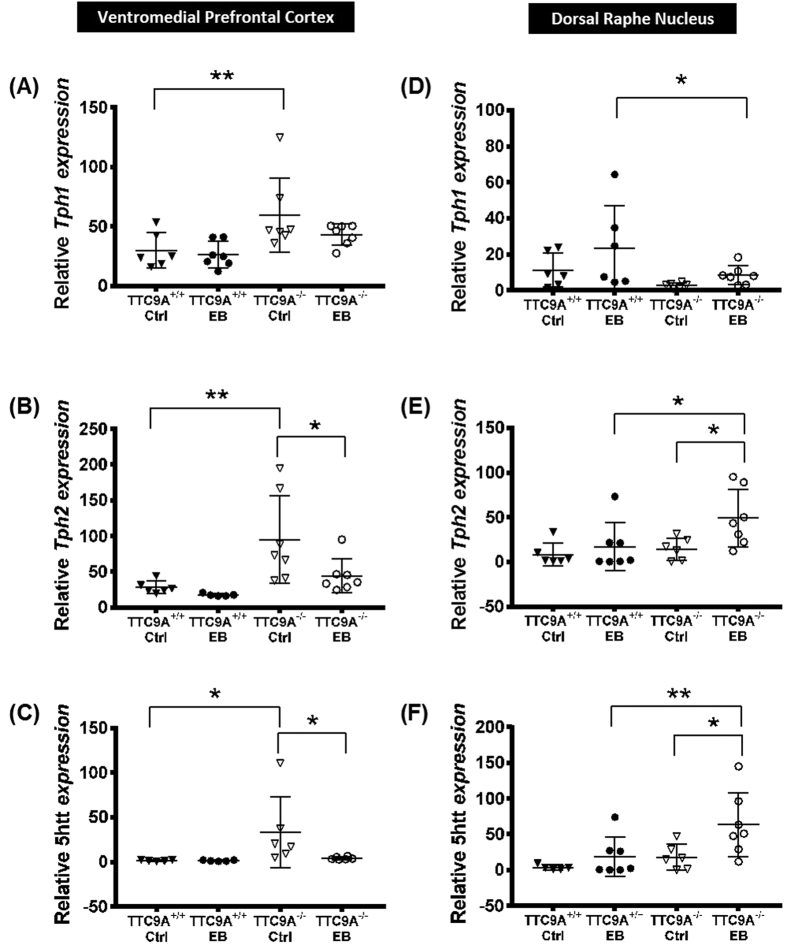
Effects of TTC9A^−/−^ on TPH and 5-HTT expression in the vmPFC and DRN. Relative mRNA expressions of TPH1 (**A**,**D**), TPH2 (**B**,**E**), and 5-HTT (**C**,**F**) in the ventromedial prefrontal cortex (vmPFC) and dorsal raphe nucleus (DRN) of TTC9A^−/−^ and TTC9A^+/+^ ovariectomized mice that received either 17-β-estradiol benzoate (EB) or CTRL treatment. Note: TTC9A^−/−^ with EB injection down-regulated TPH2 and 5-HTT in the vmPFC, while up-regulated TPH2 and 5-HTT in the DRN as compared to the TTC9A^−/−^ OVX-CTRL treated animals. The data were presented as mean ± S.E.M. Indication: *Represents *p* < 0.05, **Represents *p* < 0.01.

Two-way ANOVA of the gene expression in vmPFC, revealed a significant difference in genotype for TPH1 (F_(1,23)_ = 10.14, P = 0.004) and TPH2 (F_(1,21)_ = 10.68, *p* = 0.004), and significant difference in treatment for TPH2 (F_(1,21)_ = 4.64, *p* = 0.043). Post-hoc analysis revealed significantly higher levels of TPH1, TPH2, and 5-HTT mRNA in the vmPFC of TTC9A^−/−^ OVX-CTRL animals (5-HTT: *p* < 0.05; TPH1 & TPH2: *p* < 0.01) as compared to the TTC9A^+/+^ OVX-CTRL group (Fig. 6A–C). Furthermore, EB treatment reduced significantly the expression of TPH2 and 5-HTT, but not TPH1, in TTC9A^−/−^ mice (*p* < 0.05; Fig. 6B,C). On the other hand, EB treatment had no effect on the expression of TPH1, TPH2 and 5-HTT in TTC9A^+/+^ mice.

In the DRN, two-way ANOVA revealed significant difference between genotypes for TPH1 (F_(1,21)_ = 5.42, *p* = 0.030) and 5-HTT (F_(1,21)_ = 6.26, *p* = 0.021), and significant difference between treatments for TPH2 (F_(1,22)_ = 5.509, *p* = 0.028) and 5-HTT (F_(1,21)_ = 6.53, *p* = 0.018). Post-hoc analysis revealed significantly higher levels of TPH2 and 5-HTT mRNA in OVX-EB TTC9A^−/−^ mice compared to TTC9A^−/−^ OVX-CTRL mice (*p* < 0.05; Fig. 6E,F). Furthermore, there were significantly higher levels of TPH2 and 5-HTT mRNA, and lower levels of TPH1 mRNA in OVX-EB TTC9A^−/−^ than in OVX-EB TTC9A^+/+^ mice (TPH1 and TPH2, *p* < 0.05; 5-HTT, *p* < 0.01; Fig. 6D–F).

## Discussion

While TTC9A mRNA expression was highly detected in the brain, its function with estrogen in the regulation of mood and anxiety behavior has not been reported. The findings from the present study demonstrated that TTC9A^−/−^ induced anxiogenic behavior with significant increase of escape latency in the home-cage emergence test and increased immobility in the open-field test in the female, but not in the male, mice. Although we observed comparable escape latencies for male TTC9A^+/+^, male TTC9A^−/−^, and female TTC9A^−/−^ in the home-cage emergence test, we cannot interpret that both the TTC9A^+/+^ and TTC9A^−/−^ male mice were highly anxious as other behavioral tests showed no significant difference in their exploratory and anxiety-related behaviors. Furthermore, most rodent models demonstrate lower anxiety-like behavior in females, compared to males in the light-dark box test^26^, elevated plus-maze^27^, and elevated t-maze tests^28^. Given that there were no major differences between the TTC9A^+/+^ and TTC9A^−/−^ male mice, and we argue that TTC9A^−/−^ induced anxiogenic behavior is specific to female mice.

Our observation of anxiogenic behavior in female TTC9A^−/−^ mice was further supported by a remarkable decrease of exploratory rearing behavior in both the novel-cage exploration and rat-cage behavior tests, as well as increase of immobility behavior in the open-field and rat-cage behavior tests. In addition, there was also a significant decrease in the social behavior of the TTC9A^−/−^ female mice. It is of note that while we found no remarkable difference in the time-spent of animals in the center zone of the open-field, we showed a significant increase of immobility and time-spent in the corner zone in open-field for TTC9A^−/−^ female mice, which would indicate anxiety-like behavior. A possible explanation for this phenomenon could be due to a ‘minimal’ or ‘low’ level of anxiety response induced in the TTC9A^−/−^ female mice which could possibly result in no significant changes in the open-field center zone duration. This observation is strongly supported by our previous studies in which we found no significant difference in the center zone of the open-field after serotonergic drugs treatment (e.g. buspirone, escitalopram) in a low-anxiety condition as compared to the high-anxiety condition^25^,^29^. Regardless, our findings provide evidence that TTC9A gene has an important role in modulating the anxiety-like behavior.

Reports have suggested that in peri- and post-menopausal period, the incidence of mood and anxiety disorder is relatively higher in women due to estrogen deficiency^3^. Estrogen replacement therapy has shown a beneficial effect in the peri- and post-menopause women in clinical studies including randomized double-blind trials^20^,^30^. Similarly, anxiety- and depressive-like behaviors can also be found in animal models with OVX, and this effect can be reversed by administration of EB regimen^31^,^32^,^33^. This behavioral alteration in female animals with reduced estrogen after OVX is generally considered a well-validated animal model for human post-menopausal mood-related disorder^34^,^35^. Consistent with the reported studies, EB treatment reduced the escape latency in TTC9A^+/+^ OVX mice. However, we observed no depressive-like behavior in the tail-suspension test in the TTC9A^+/+^ OVX-CTRL mice. This could be due to the short duration of EB treatment. Reports have suggested that some effects on behavior can only be observed several months after ovariectomy^36^,^37^.

Since our previous data suggest that TTC9A negatively regulates estrogen action^15^,^21^, we investigated the effect of estrogen on the behaviors of TTC9A^−/−^ mice following OVX. The anxiety-like behavior in TTC9A^−/−^ mice following ovariectomy was generally similar to the TTC9A^+/+^ mice except for the decrease of the escape latency in TTC9A^−/−^ mice in the home-cage emergence test. This suggests some functional interaction between TTC9A and estrogen, which is supported by the evidence, that TTC9A^+/+^ mice exhibited decrease in anxiety in the home-cage emergence test in response to EB, and that TTC9A^−/−^ mice showed increased anxiety in both the home-cage emergence test and in the tail-suspension test after EB treatment. This implies that TTC9A modulates estrogen action on anxiety and depressive behaviors such that its absence leads to estrogen to exert an anxiogenic effect.

The serotonergic system is important in mood and anxiety disorders and effective antidepressant therapy commonly uses drugs that increase the extracellular concentration of 5-HT^6^,^38^. Serotonergic neurons are found in the DRN and project their fibers to the forebrain regions including the prefrontal cortex and limbic structures^39^,^40^. TPH1 and TPH2 are rate limiting enzymes for 5-HT synthesis and it has been reported that estrogen increased the expression of TPH1 and TPH2 in the DRN and this regulation is mediated by ERβ through an estrogen response element in the 5′ untranslated region^22^,^41^. Our results show a trend of increased TPH1, TPH2 and 5-HTT expression in DRN in response to EB but the observation is not statistically significant. TTC9A gene deletion significantly increased the expression of TPH2 and 5-HTT in the DRN in response to EB. EB treatment also inhibited the expression of TPH2 and 5-HTT in vmPFC of TTC9A^−/−^ mice and this is not observed in TTC9A^+/+^ mice. In addition, TTC9A^−/−^ OVX mice expressed higher levels of TPH1, TPH2 and 5-HTT in vmPFC than TTC9A^+/+^ OVX mice. Together, TTC9A appears to influence estrogen action on gene expression such that in its absence, estrogen is more active in repressing (in the case on vmPFC) or activating (in the case on DRN) the genes related to 5-HT synthesis or transmission.

Concerning the 5-HT mediated regulation of anxiety, there are several discrepancies of its gene expression within the DRN and vmPFC for mood and anxiety-like behaviors. However, our results corroborate previous findings that showed increased 5-HTT expression in the DRN^42^, and reduced TPH synthesis and 5-HTT function in the vmPFC in animal models of depression^8^,^9^. Furthermore, the increase of TPH^43^,^44^, with lower levels of 5-HT and its metabolite (5-hydroxyindoleacetic acid) in the DRN (or brainstem), as well as decreased 5-HTT binding in the ventral PFC, have also been reported in patients with major depression and suicidal behaviors^44^,^45^,^46^. The plausible explanation for an increase of 5-HTT and TPH expression in the DRN of anxiogenic mice could be that it resulted from the upregulation of homeostatic responses to the deficiency in 5-HT release, dysfunctional receptor activation, and/or hypoactive 5-HT synthesizing enzymes^43^,^44^,^46^. Nonetheless, the involvement of serotonergic system for anxiety-like behaviors is complex due to different existing neuronal subpopulations in the DRN and their multi-synaptic interactions. Nevertheless, the effects of TTC9A^−/−^ on estrogen regulation of TPH1, TPH2 and 5-HTT which influences anxiety-like behaviors will remain an interesting topic for future investigation.

In conclusion, our study showed for the first time that absence of TTC9A gene induced anxiety-like behavior in mice, and this response was substantially abolished after ovariectomy. We confirmed that TTC9A^−/−^ OVX mice exhibited significantly higher level of anxiety and behavioral despair in response to EB than TTC9A^+/+^ OVX mice. In addition, TTC9A^−/−^ heightened EB inhibition of TPH1, TPH2 and 5-HTT in vmPFC and strengthened EB induction of TPH2 and 5-HTT in DRN. Taken together, these findings provide novel insight into the role of TTC9A on estrogen modulation in the mood and anxiety disorders.

## Methods

### Subjects

5 month-old mice were socially housed in the individual-ventilated cages on corncob bedding under controlled temperature (about 24–26 °C), humidity (60–70%), and 12/12-h light/dark cycle (lights off at 19:00 h). Food and water were available *ad libitum*. All experimental procedures and methods were performed in accordance with the relevant guidelines and regulations, and approval from the Institutional Animal Care and Use Committee (IACUC), Nanyang Technological University, Singapore (Reference Number: ARF-SBS/NIE-A 0169 AZ).

### Generation of TTC9A knockout mice

The detailed description of generation of the TTC9A^−/−^ mice has been previously described^15^,^21^. In brief, the TTC9A targeting vector was generated by replacing the TTC9A exon 1 with a loxp flanked neomycin cassette, and electroporated into R1 mouse ES cells. The TTC9A heterozygous ES clones were microinjected into 8 cell stage mouse embryos isolated from C57BL/6J. The TTC9A chimeras were crossed to C57BL6/6J mice to generate TTC9A heterozygous mice. These TTC9A heterozygous mice were crossed to obtain the TTC9A homozygous mice.

### Experimental design

Two systematic behavioral studies were used to evaluate the mood - and anxiety-related behaviors, and memory function in both the male and female TTC9A^−/−^ and TTC9A^+/+^ mice. In behavioral study 1, home-cage emergence test, novel-cage exploration test, and rat-cage behavior test were used to measure the anxiety and rearing behaviors, object recognition test was applied to measure the short- and long-term memory functions; sucrose preference test was used to measure hedonic activity; and finally forced swimming test was used to assess behavioral despair (Fig. 2A). Throughout the behavioral experiments, estrous cycle was determined by vaginal swab cytology and only females in diestrus stage were included in the analysis.

### Drug administration

17-β-Estradiol benzoate (EB; Sigma-Aldrich, Missouri, USA) was dissolved in the sesame oil with 0.25% benzyl alcohol and injected subcutaneously at a dosage 10 μg/kg in both the TTC9A^−/−^ and TTC9A^+/+^ female mice. The selection of this dosage was based upon previous studies of dose-dependent effects of EB treatment on anxiety level in OVX rodents which producing physiological plasma levels of estradiol and anxiolytic behavior 48 h later^47^,^48^. The behavioral testing was conducted in accordance with the order of time after EB injection.

In behavioral study 2, a new set of female mice were subject to OVX procedure followed by treatment of either control (CTRL; injection of sesame oil with 0.25% benzyl alcohol) or estradiol benzoate (EB). Firstly, mice were assessed for their anxiety and rearing behaviors using the open-field test, novel-cage exploration test, social interaction test, and sucrose preference test. Ten days after recovery from OVX surgery, all animals were injected with either CTRL or EB, and subjected to behavioral testing on day 20 and 21. In this paradigm, mice were assessed for anxiety-related behaviors in the elevated plus-maze (24 h), cylinder test (26 h), and home-cage emergence test (48 h), and finally a tail-suspension test (52 h) was used to measure behavioral despair. Behavioral testing conducted 24 h and 48 h after EB injection were based on previous studies demonstrating its promising effects on the behavioral responses and EB-induced synaptic plasticity changes^47^,^48^,^49^. The experimental design is presented in a schematic representation in Fig. 4A.

### Surgical procedures

A detailed description of the surgical procedure has been previously reported^50^. In brief, the animals were ovariectomized and anesthetized using a combination of ketamine (75 mg/kg) and xylazine (5 mg/kg) injected subcutaneously. After surgery, animals had a 10-day recovery period.

### Behavioral testing and evaluation

All testing and analysis of animal behaviors were performed by researchers who were blind to the treatments and condition of testing. The tests for behavioral study 1 were conducted during 20:00–00:00, while the tests for behavioral study 2 after OVX were performed in the time order of 24 h, 26 h, 48 h, 52 h after injections during 20:00–01:00. Mice were given at least 30 min acclimatization in the room before testing. After each test the arena or set-up was cleaned with 70% ethanol and dried before the next mouse was tested.

#### Home-cage emergence test

The procedure of home-cage emergence test was performed as previously described with minor modifications^51^. In brief, a novel mouse home-cage was placed on a platform and the lid of the home-cage was removed. A grid was placed over the edge of the home-cage in order to make it easier for the mouse to leave the home-cage. The experimenters measured the escape latency (time taken for the mouse to climb out of its cage onto the grid). If the mouse did not escape from its home-cage within 10 min, the session was ended, and the mouse was given a score of 600 s.

#### Novel-cage exploration test

The standard novel-cage exploration test was performed as previously described^52^. Mice were introduced into a standard plastic cage filled with fresh corncob bedding. The number of exploratory rearing was counted during a 5 min period by offline recorded video observation.

#### Rat-cage behavior test

The test was conducted in a standard rat cage (25 × 35 × 25 cm), with an open top and a dark floor. The behavior of each mouse was video recorded. The duration of immobility and frequency of rearing were analyzed for the total 5 min observation of the mice spent in the arena.

#### Object recognition test

The object recognition test was performed as previously described with minor modifications^53^. The test consists of a habituation phase, acquisition phase, and finally the test phases for short-term memory (1 h after acquisition phase), and long-term memory (24 h after short-term memory test) functions. In the habituation phase, mice explored an open-field arena (40 × 40 × 40 cm^3^) for 10 min. Next day, during acquisition phase, mice were presented with two identical objects (object A + object A) in the same open-field environment for a total duration of 3 min. After 1 h, mice were tested in the same arena with one familiar object and one novel object (object A + object B) in the short-term memory phase. On the next day (24 h later), the familiar object was replaced by another novel object in the long-term memory test phase (object C + object B). The positions of objects were counter-balanced across subjects and testing phase. The duration spent on exploration in each object was video recorded. The total duration of exploration time was considered as the sum of time spent at both objects. *Discrimination index* was calculated as follows, the exploration time (T) of the novel object minus the familiar object, then dividing this value by the total amount of exploration of the novel and familiar objects [DI = (T_N_ − T_F_)/(T_N_ + T_F_)]^54^.

#### Sucrose preference test

The sucrose preference test was performed as previously described with minor modifications^51^,^55^. One day prior to the test, animals were exposed to drink 1% sucrose solution and water for 1 h, and subsequently followed by food and water deprivation for 14 h. After 14 h fasting, mice were again given a choice between two bottles of 1% sucrose solution and water, and tested for the total duration of 1 h. To prevent side preference for water or sucrose consumption, the two bottles were counter-balanced during the pre-test and test sessions. The sucrose and water intake were calculated from the total amount sucrose solution or water consumed (ml) by weighing the bottle pre- and post-testing session. The relative sucrose consumption was corrected for body weight of the animals (ml/kg). The sucrose preference was calculated as a percentage of the sucrose consumption with respect to the total amount of liquid intake. The animals were tested individually during the sucrose consumption, and they were placed back into their respective group-housing cage immediately after the test.

#### Forced swimming test

The testing was carried out using a transparent Plexiglas cylinder (height 30 cm × diameter 10 cm). The cylinder was filled with tap water (25 ± 1 °C) to a depth of 10 cm. The testing procedure was performed over two consecutive days as previously described^51^,^56^. In a pre-test session, each mouse was placed in the water for 15 min. The following day, the mouse was again tested in the cylinder containing water for 10 min and the behavior was recorded by a digital camera. The duration of the following behaviors was measured: “*immobility*” (no movements or small and infrequent movements performed solely to maintain the nose above the water), “*swimming*” (active swimming with the forepaws), and “*climbing*” (scratching of cylinder walls using both forepaws and hind paws).

#### Open-field test

The test was conducted in an enclosed square, made of white plastic box (40 × 40 × 40 cm^3^). Behavioral testing started after the mouse had been individually placed in the middle of the arena. A trial was stopped after 5 min and the behavior of each mouse was video recorded. The duration of immobility and time-spent in the different zones (center, periphery and corner) of the open-field were measured and analyzed as previously described with minor modifications^29^,^57^.

#### Social interaction test

The social interaction behavior was conducted in a Plexiglas rectangle box (25 × 35 × 25 cm) with a wire net (30 × 40 cm) as a separator between the two animals. Animals were allowed to freely interact in the arena and the time-spent interacting was video recorded^58^.

#### Elevated plus-maze test

The apparatus consisted of two open-arms (40 × 5 cm) and two closed-arms (40 × 5 × 10 cm) that extended from a common central platform (5 × 5 cm). The apparatus was elevated at a height of 50 cm from the floor. The mouse was placed in the central platform and individually tested for 5 min. The duration of time-spent and frequency of entry into the open- and closed-arms were measured.

#### Cylinder test

All mice were tested in the transparent Plexiglas cylinder (height 30 cm × diameter 10 cm) for a total of 5 min duration. The behavior of each mouse was video recorded and the frequency of rearing was analyzed.

#### Tail-suspension test

All mice were subjected to the tail-suspension procedure as previously described^59^ for a total duration of 5 min. The mice were suspended by hanging their tails with adhesive tape in an individual compartment of 40 cm from the floor. The duration of immobility was measured when mice were completely motionless.

### Southern blotting analysis and polymerase chain reaction (PCR) genotyping

The genomic DNA from ES cells or tail biopsies was digested with Sca I, separated on a 0.8% agarose gel and blotted onto a positively charged nylon membrane overnight. These membranes were then hybridized with the digoxigenin (DIG)-labelled 5′ probe overnight, washed, then blocked with blocking buffer and finally probed with anti-DIG-alkaline phosphatase antibody at a 1:5000 dilution. These nylon membranes with digoxigenin-labelled nucleic acids were detected using chemiluminescent immunodetection methods. PCR was also used for routine mouse genotyping and the following PCR conditions and primer pairs were used for this purpose: 94 °C (30 sec), 60 °C (30 sec), 72 °C (1 min) for 30 cycles and a final extension step at 72 °C (7 min). TTC9A allele primers: TTC9A-FP: 5′-CACACGAGTTCAAGAGCCAAGGG-3′; TTC9A-RP: 5′-GCTTCAACCGTCTTGCTCTG-3′. *Neomycin* primers: Neo-FP: 5′-ACTGGGCACAACAGACGATCGG-3′; Neo-RP: 5′- GGAAGCGGTCAGCCCATTCG-3′.

### Real-time PCR analysis

After behavioral study 2, all mice were sacrificed and their brains were harvested for real-time PCR analysis. The vmPFC and DRN were dissected in the cryostat CM3050 (Leica Microsystems, Wetzlar, Germany) according to the anatomical region of the Mouse Brain Atlas by Franklin and Paxinos, 2007. The brain regions of interest, namely the dorsal raphe nucleus and ventromedial prefrontal cortex were homogenised in cold TRIzol (Invitrogen, Carlsbad, USA) and total RNA was extracted using TRIzol reagent, chloroform:isoamyl-ethanol (24:1) and phenol:chloroform:isoamyl-ethanol (50:24:1; Sigma-Aldrich, St. Loius, USA), precipitated using isopropanol and washed with 75% ethanol in DEPC-treated water (Sigma-Aldrich, St. Loius, USA) before re-suspending in 0.2% DEPC-treated water. From this 2 μg of total RNA was reverse transcribed using Superscript II reverse transcriptase (Invitrogen, Carlsbad, USA). Real-time PCR was performed with KAPA SYBR^®^ FAST qPCR Master Mix (Kapa Biosystems, Wilmington, USA) on an ABI Prism 7000 sequence detection system (PE Applied Biosystems, Foster City, CA). The real-time PCR primers used were: tryptophan hydroxylase (TPH) 1 (forward 5′-GGGCTTGACTTTGTCTCTGC′ and reverse 5′-GTTTGAATCTGGCCTGGTGT-3′), TPH2 (forward 5′-CCTACACGCAGAGCATTGAA-3′ and reverse 5′-CTAGGCATCAAATCCCCAGA-3′), serotonin (5-hydroxytryptamine; 5-HT) transporter (5-HTT) (forward 5′-CTTCAGCCCCGGATGGTT-3′ and reverse 5′-GTGGACTCATCAAAAAACTGCAA-3′), and Glyceraldehyde 3-phosphate dehydrogenase (GADPH) (forward 5′-GTCGGTGTGAACGGATTTG-3′ and reverse 5′-AATTTGCCGTGAGTGGAGTC-3′). Fold changes were calculated from the Ct values and the relative expression levels of the TPH1, TPH2, and 5-HTT were determined by normalizing their Ct values against GADPH Ct values.

### Statistical analysis

Statistical analysis were performed using GraphPad Prism 7. All data are presented as mean ± S.E.M. Tests done in behavior study 1 were analyzed using independent sample t-tests. A repeated-measures analysis was used to measure the within- and between-subject components in the object recognition test. Tests done before EB injection in behavior study 2 were analyzed using independent sample t-tests. Tests done after EB injection were analyzed using a two-way ANOVA with LSD post-hoc test. Real-time PCR results were analyzed using two-way ANOVA with LSD post-hoc. All *p*-values < 0.05 were considered significant. Outliers were identified and removed using ROUT method.

## Additional Information

**How to cite this article**: Lim, L. W. *et al*. Tetratricopeptide repeat domain 9A modulates anxiety-like behavior in female mice. *Sci. Rep*. **6**, 37568; doi: 10.1038/srep37568 (2016).

**Publisher’s note**: Springer Nature remains neutral with regard to jurisdictional claims in published maps and institutional affiliations.

## Figures and Tables

**Figure 1:**
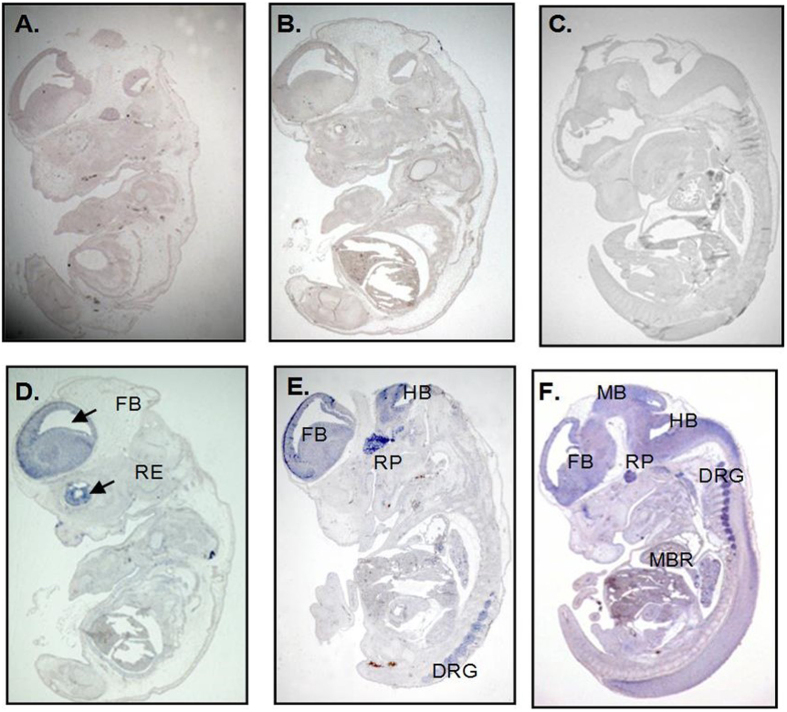
Visualization of TTC9A expression in the developmental embryonic stage. TTC9A expression in TTC9A^+/+^ mice embryo day 13.5 visualized by RNA-ISH. *In-situ* hybridization with the control probe (**A**–**C**) and with the TTC9A probe (**D**–**F**). The parasagittal lateral (**A**,**D**) section through the embryo at 13X magnification power show TTC9A expression in the forebrain (FB) and retina (RE), and parasagittal medial section (**B**,**E**) show TTC9A expression in the forebrain (FB), hindbrain (HB), Rathke’s pouch (RP) and the dorsal root ganglion (DRG). The E13.5 mid sagittal section shows TTC9A mRNA expression at the forebrain (FB), hind brain (HB), mid brain (MB), Rathke’s pouch (RP), dorsal root ganglion (DRG), and around the main bronchus (MBR).
